# Effect of EDTA and Zero-Valent Iron Nanoparticles on Phytoremediation Capacity of *Cistanthe grandiflora*

**DOI:** 10.3390/plants15081183

**Published:** 2026-04-12

**Authors:** Andrea Lazo, Pamela Lazo, Henrik K. Hansen, Alejandro Zambra, Waldo Pérez, Arnold Solano

**Affiliations:** 1 Departamento de Ingeniería Química y Ambiental, Universidad Técnica Federico Santa María, Avenida España 1680, Valparaíso 2390123, Chile; henrik.hansen@usm.cl (H.K.H.); alejandro.zambra@sansano.usm.cl (A.Z.); asolano@usm.cl (A.S.); 2 Instituto de Química, Universidad de Valparaíso, Avenida Gran Bretaña 1111, Valparaíso 2360102, Chile; pamela.lazo@uv.cl; 3 Centro de Observación Marina para Estudios de Riesgos del Ambiente Costero (COSTA-R), Universidad de Valparaíso, Valparaíso 2540006, Chile; 4 Hochschule Burgenland, University of Applied Sciences, Campus Pinkafeld, Steinamangerstraße 21, A-7423 Pinkafeld, Austria; waldo.fpf@gmail.com

**Keywords:** phytoremediation, tailings, nanoparticles, EDTA

## Abstract

Mining activities in Chile generate massive amounts of tailings, creating significant environmental risks due to heavy metal contamination. Phytoremediation offers an eco-friendly solution, yet studies on native Chilean species are scarce. This study evaluates the effects of ethylenediamine tetraacetic acid (EDTA) and nanoscale zero-valent iron (nZVI) on the potential of the native *Cistanthe grandiflora* for the phytoremediation of copper mine tailings. A six-month pot experiment was conducted with four treatments: EDTA 300 mg·kg^−1^, EDTA 600 mg·kg^−1^, nZVI 500 mg·kg^−1^, and a control group without additions. The results indicate that *Cistanthe grandiflora* primarily acts as a phytostabilizer, accumulating higher metal concentrations in roots than in aerial parts. The application of EDTA significantly enhanced the Bioconcentration Factor for Cu, Ni, Pb, and Mo, increasing BCF values from 0.5 to 1.0 or more in several cases. Specifically, a lower dose of EDTA (300 mg·kg^−1^) successfully increased the Translocation Factor (TF) of cadmium to 1.3, suggesting a potential for phytoextraction for this element. Conversely, nZVI application showed a limited impact, slightly improving the Translocation factor for copper and chromium but without exceeding unity. These findings demonstrate that *Cistanthe grandiflora*, assisted by EDTA, is a promising candidate for the phytostabilization of heavy metals in mine tailings.

## 1. Introduction

Soil pollution frequently originates from anthropogenic sources, including agriculture and industrial activities such as mining, smelting, refining, and manufacturing [1,2]. This contamination poses indirect risks to both humans and animals [3]. Specifically, mine tailings represent a significant source of soil and water contamination. These residues, generated by mining processing plants, consist of fine-grained materials with variable water content. This composition facilitates chemical reactions, the dissolution of toxic substances, and acid generation, creating an inherent risk to surrounding soil and groundwater ecosystems.

Heavy metals represent a major environmental pollutant due to their non-degradable nature and potential to generate leachate. Based on biological requirements, metals and metalloids are classified as essential (e.g., Co, Cu, Ni, and Zn) and non-essential (e.g., As, Cd, Hg, and Pb) for biota [4].

Given the absence of specific legislation regulating soil contaminant levels in Chile, preventing contamination and reducing or stabilizing existing pollutant concentrations is essential. Therefore, identifying cost-effective and restorative remediation alternatives is crucial for achieving environmental sustainability. Furthermore, considering the region’s current water scarcity, implementing techniques that minimize water usage is paramount. In this context, restorative techniques such as phytoremediation emerge as a promising alternative. Plant species with low water requirements, such as *Cistanthe grandiflora*, can effectively contribute to the decontamination of sites polluted with heavy metals and metalloids.

Phytoremediation is an eco-friendly technique that utilizes plants to remove, degrade, or neutralize pollutants in soil, water, and air [5]. Currently, several phytoremediation technologies are successfully utilized for cleaning up contaminated environments: phytoextraction involves plants absorbing contaminants (such as heavy metals) through their roots and accumulating them in their aerial tissues [6]; phytodegradation entails the breakdown of organic pollutants into less harmful substances [7]; rhizodegradation is the breakdown of contaminants by microorganisms in the rhizosphere [8]; phytostabilization refers to the immobilization of contaminants in the soil, thereby reducing their mobility and bioavailability [9]; and finally, phytovolatilization relies on the ability of certain plants to absorb volatile contaminants and release them into the atmosphere in a less toxic form [10].

Plants are classified as hyperaccumulators based on two main criteria. According to Krämer et al. [11], a key indicator is the heavy metal concentration in the aerial parts of the plant, typically ranging between 1000 and 10,000 mg·kg^−1^, depending on the specific metal. For highly toxic metals like Cd, lower concentrations—even as low as 100 mg·kg^−1^—are considered indicative of hyperaccumulation. Another criterion, established by Baker et al. [12], is the translocation factor (TF), defined as the ratio of element concentration between the aerial parts and the roots. A TF value above 1 indicates that the contaminant is primarily accumulated in the shoots, classifying the plant as a hyperaccumulator [12]. Additionally, the Bioconcentration Factor (BCF) is used to describe the ability of plants to accumulate elements from the substrate, while the TF assesses the potential for successful upward translocation [13]. BCF values greater than 1 are indicative of a plant species’ potential for successful phytoremediation [13,14,15].

Phytoremediation is often enhanced by the addition of amendments to improve substrate characteristics. In the case of mine tailings—which inherently lack essential nutrients for proper plant growth and development—the addition of nutrients, chemical amendments, and/or organic matter is critical for achieving plant self-sustainability over time [16,17,18,19,20].

To enhance phytoremediation efficiency across various plant species, several assisted techniques have been explored. Two prominent methods involve nanotechnology and the application of chelating compounds (synthetic or organic).

Regarding nanotechnology, its environmental applications have expanded significantly in recent years. Nanomaterials possess a strong affinity for metals due to their small size and large surface area, allowing them to readily penetrate the contamination zones of metal-stressed habitats [21]. Their large surface area-to-volume ratio enhances their reactivity in chemical or biologically mediated remediation reactions [22]. Available data also indicate that nanomaterials can alleviate abiotic stress-induced damage by activating the antioxidant defense system of plants [21]. Moreover, nanomaterials can absorb, adsorb, and reduce environmental contaminants. A prime example is the use of nanoscale zero-valent iron (nZVI) to reduce the concentration and toxicity of specific pollutants [23].

Both nZVI and iron oxide nanoparticles have shown great promise in improving the uptake and degradation of heavy metals and organic pollutants [24,25,26]. In the context of heavy metals, iron nanoparticles facilitate phytoremediation through several mechanisms: (1) Reduction of toxic metal ions to less toxic forms, aiding in their immobilization and reducing bioavailability [27]; (2) Enhancement of metal solubility, making certain metals more available for plant uptake and subsequent translocation to harvestable biomass [6]; and (3) Soil modification, as the application of iron nanoparticles can alter soil pH and structure [28].

Recent studies have demonstrated that while low nZVI dosages positively affect plant growth, high dosages can induce toxicity [29,30]. Consequently, nZVI application directly influences a plant’s capacity for metal accumulation. For instance, Gong et al. [31] reported a positive effect of low-dose stabilized nZVI on Cd phytoremediation, and Huang et al. [32] observed a stimulatory effect on *Lolium perenne* exposed to Pb. Conversely, Wang et al. [25] indicated a suppressive effect on Cr uptake following nZVI application in rape (*Brassica campestris* L.) and Chinese cabbage (*Brassica pekinensis*). Notably, there remains a paucity of studies regarding the effects on nZVI on native and endemic species.

Research indicates that the combined use of phytoremediation, chemical amendments, and nZVI maximized remediation benefits while minimizing nanoparticle toxicity, offering new insights into the interactions between zero-valent iron and metalloids [33].

Another successful strategy is chelate-assisted phytoextraction. This method enhances the ability of both accumulator and non-accumulator species to extract contaminants by increasing metal bioavailability, thereby facilitating root uptake and shoot translocation [2,34].

Among organic chemical amendments, ethylenediamine tetraacetic acid (EDTA) is widely recognized as the most efficient organic ligand for enhancing metal uptake, solubilization, and translocation due to its ability to form highly stable and soluble metal–EDTA complexes [35,36,37,38].

The impact of EDTA on heavy metal accumulation varies significantly depending on the plant species, the target metal, and the soil type, occasionally resulting in accumulations up to 200-fold higher than untreated controls [34]. For example, in a 24-h experiment, lead accumulation increased fourfold in the *Sedum alfredii* roots and twofold in *Vicia faba* seedlings [35]. Another study on the assisted phytoremediation of heavy metals using *Typha latifolia* and *Chrysopogon zizanioides* demonstrated that the co-application of EDTA and Al_2_(SO_4_)_3_ significantly improved both metal accumulation and translocation factors [39]. To date, no studies have been published regarding the use of EDTA in phytoremediation of native or endemic Chilean flora.

While both nZVI and EDTA are utilized to enhance phytoremediation, their mechanisms of action are fundamentally contrasting. On the one hand, nZVI primarily acts to immobilize heavy metals in the substrate through processes such as chemical reduction, adsorption, and co-precipitation, thereby reducing their bioavailability and mitigating environmental toxicity [23,27]. On the other hand, EDTA functions as a powerful chelating agent that significantly enhances metal mobility and solubility by forming highly stable metal–EDTA complexes in the soil solution [35,37]. This chelation process facilitates the root uptake and subsequent upward translocation of contaminants [34]. Understanding these opposing mechanisms—immobilization versus mobilization—provides a critical theoretical foundation for interpreting how each amendment uniquely influences the plant’s phytoremediation strategy, determining whether it shifts towards assisted phytostabilization or phytoextraction.

Despite the widespread investigation of assisted phytoremediation technologies, studies focusing on native Chilean species remain remarkably scarce. To date, existing research has primarily focused on non-native hyperaccumulator species or the remediation of single heavy metals. Consequently, the remediation mechanisms of salt- and metal-tolerant species endemic to the arid mining regions of Chile remain critically underexplored. Addressing this knowledge gap is essential, as employing native metallophytes like *Cistanthe grandiflora* offers a highly sustainable, ecologically compatible, and novel approach for the stabilization of multi-metal contaminated mine tailings. Building upon previous findings [40,41], this study assesses the independent effects of EDTA and nZVI on the Bioconcentration Factor (BCF) and Translocation Factor (TF) of this endemic species, ultimately determining their potential for assisted phytostabilization or phytoextraction in copper mine tailings.

## 2. Materials and Methods

### 2.1. nZVI Synthesis Procedure

The synthesis of nanoscale zero-valent iron (nZVI) particles was performed using the sodium borohydride reduction method. This technique involves reducing iron (III) to zero-valent iron using sodium borohydride in a three-necked flask reactor equipped with a variable-speed mechanical stirrer.

For the synthesis, a 0.33 M iron(III) chloride solution was prepared by dissolving 5.406 g of FeCl_3_·6H_2_O in a 4:1 (*v*/*v*) ethanol/water mixture (48 mL of ethanol and 12 mL of deionized water). The iron solution was added to the reactor and stirred at 200 rpm under a continuous flow of N_2_ gas. A separate 0.5 M borohydride solution, prepared by dissolving 3.783 g of NaBH_4_ in 200 mL of deionized water, was then added dropwise to the flask at a rate of 0.2 mL·s^−1^ using a burette.

Upon completion of the borohydride addition, the mixture was stirred for an additional 15 min. The resulting nZVI particles were collected by vacuum filtration and washed three times with deionized water (approximately 100 mL) and three times with absolute ethanol (approximately 100 mL). The synthesized particles were subsequently dried at 323 K for 24 h and stored at 277 K under a thin layer of ethanol.

### 2.2. Plant and Tailings

The experiments were conducted using *Cistanthe grandiflora*, commonly known as “Pata de guanaco” or “Doquilla”. This succulent perennial is endemic to Chile, belongs to the *Portulacaceae* family, and is geographically distributed between the Antofagasta and Ñuble regions.

Approximately 550 kg of tailings were collected from the Minera Las Cenizas facility (32°28′16.1″ S, 71°05′00.2″ W), which has a projected capacity of 3 million tons of paste tailings. The collected material underwent a specific preparation process: first, it was dried at 105 °C to a constant weight. Subsequently, the tailings were ground and homogenized using a ball mill and passed through an ASTM N°18 mesh.

The prepared material was then characterized. The pH was determined using EPA Method 9045D, and elemental analysis was conducted via inductively coupled plasma-optical emission spectroscopy (ICP-OES) on a Optima 2000V (PerkinElmer, Inc., Shelton, CT, USA), with a detection limit of 0.005–0.01 ppm.

For the experimental trials, pots were prepared with a mixture of 3.2 kg of processed tailings and 0.8 kg of leaf mold. *Cistanthe grandiflora* seedlings, approximately 10 cm in height, were transplanted into each pot. The plants were allowed to acclimate for two weeks before treatment application. The experiment consisted of four treatments, each with six replicates, detailed as follows:

○ Treatment 1: 300 mg of EDTA per kg of dry substrate.
○ Treatment 2: 600 mg of EDTA per kg of dry substrate.
○ Treatment 3: 500 mg nZVI per kg of dry substrate.
○ Treatment 4: Substrate consisting only of tailings and leaf mold, without the addition of nZVI or chelating agents.

For treatments 1 and 2, EDTA was applied as an aqueous solution. For Treatment 3, the nZVI was applied as a suspension in distilled water containing sodium dodecyl sulfate (SDS). All amendments were administered at the onset of the experiment. During the six-month growth period, each potted plant received a weekly dose of water (30 mL) and a monthly dose of commercial organic stimulant made from the alga *Ascophyllum nodosum* (5 mL). The plants were maintained outdoors, under a rain shelter.

At the end of the experimental period, the plants were harvested and separated into roots and aerial parts (shoots). Both plant tissue and tailing samples underwent microwave-assisted acid digestion, using an Ethos Easy system (Milestone Srl., Sorisole, Italy) following an identical protocol, differing only in sample mass (0.200 g for plant tissue and 0.060 g for tailings). Samples were placed in PTFE vessels containing 8 mL of HNO_3_ (70%) and 2 mL of H_2_O_2_ (30%). Following a 4-h pre-digestion, the microwave digestion cycle was completed. The digestate was quantitatively transferred to a 25 mL volumetric flask, diluted to volume with deionized water, and analyzed by ICP-OES for Cu, Zn, Ni, Pb, Mo, Fe, Cd, and Cr. To accurately quantify these elements, an eight-point calibration curve (0 to 8000 ppm) was generated using a Merck-certified multi-elemental standard solution [40,41].

Statistical differences were determined using a one-way analysis of variance (ANOVA) followed by Tukey’s post hoc test (*p* < 0.05).

### 2.3. Phytoremediation Factors and Removal Efficiency

To evaluate the phytoremediation process, the Translocation Factor (TF), Bioaccumulation Factor (BAF), and Bioconcentration Factor (BCF) were calculated using Equation (1), Equation (2), and Equation (3), respectively.

The TF reflects a plant’s capacity to translocate the target element from its root system to its aerial parts. A TF value below 1 suggests the plant restricts the transport of the target metal, while a value exceeding 1 indicates efficient transfer from roots to shoots. The BAF quantifies the plant’s capacity to store the target element in aerial parts relative to the concentration in the tailings. The BCF quantifies the concentration of the target element within the roots relative to the concentration in the tailings [42,43]. For BAF or BCF, values greater than 1 imply that the plant actively accumulates the target metal in its shoots or roots, respectively.

(1) $$\mathrm{TF}=\frac{\mathrm{Metal}\;\mathrm{in}\;\mathrm{the}\;\mathrm{aerial}\;\mathrm{parts}\;\mathrm{of}\;\mathrm{the}\;\mathrm{plant}}{\mathrm{Metal}\;\mathrm{in}\;\mathrm{the}\;\mathrm{roots}}$$

(2) $$\mathrm{BAF}=\frac{\mathrm{Metal}\;\mathrm{in}\;\mathrm{the}\;\mathrm{aerial}\;\mathrm{parts}\;\mathrm{of}\;\mathrm{the}\;\mathrm{plant}}{\mathrm{Metal}\;\mathrm{in}\;\mathrm{the}\;\mathrm{tailings}}$$

(3) $$\mathrm{BCF}=\frac{\mathrm{Metal}\;\mathrm{in}\;\mathrm{the}\;\mathrm{roots}\;\mathrm{of}\;\mathrm{the}\;\mathrm{plant}}{\mathrm{Metal}\;\mathrm{in}\;\mathrm{the}\;\mathrm{tailings}}$$

## 3. Results

### 3.1. nZVI Characterization

**Figure 1:**
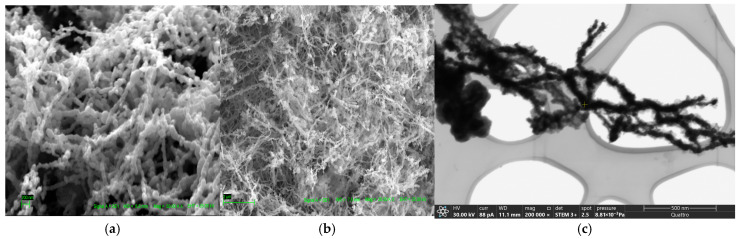
SEM images of the synthesized nZVI particles (**a**) 50,000× magnification at 25 kV, (**b**) 20,000× magnification at 25 kV, and (**c**) 200,000× magnification at 30 kV.

The SEM images of the freshly synthesized nZVI are shown in Figure 1. At 50,000× magnification, a distinct spherical morphology is resolved. The nanoparticles form chains with a linear orientation attributed to their magnetic properties [43,44,45]. Energy-dispersive X-ray spectroscopy (EDS) analysis of the synthesized nanoparticles indicates a composition of 93.75% ± 1.18% iron and 6.25% ± 1.18% oxygen. The mass percentage of Fe(0) is 80.50% ± 3.1%, Fe (II and III) is 14.1% ± 2.3%, and O is 4.9% ± 0.9%. This assumes that all measured oxygen forms Fe_3_O_4_, the most common oxidized iron species in nZVI synthesized via the borohydride method [44,45,46,47].

### 3.2. Tailing Characterization

The results of elemental analysis for tailings are presented in Table 1.

For the experimental trials, the final substrate was a mixture of 80% tailings and 20% leaf potting soil (pH = 6.2) by dry weight, resulting in a final pH of 6.7.

The final metal concentrations for each treatment in the plant tissues, along with their standard deviations, are presented in Table 2. The corresponding final metal concentrations remaining in the tailings are detailed in Table 3.

As observed in Table 2, across almost all treatments and metals (especially Cu, Zn, Cr, and Pb), the concentrations are significantly higher in the roots than in the aerial parts.

The results in Table 2 and Table 3 demonstrate a distinct impact of each treatment on the heavy metal concentrations in both the plant tissues and the surrounding tailings. The control treatment (Treatment 4) serves as the baseline for comparison, representing the natural uptake and accumulation by the plant directly from the unamended substrate.

Notably, the application of EDTA (Treatments 1 and 2) resulted in a significant accumulation of specific heavy metals in the root system compared to the unamended control. Copper and lead concentrations in the roots increased approximately 9-fold and 35-fold, respectively, demonstrating the strong mobilizing effect of the chelating agent within the substrate.

Furthermore, a distinct dose-dependent response was observed in the aerial accumulation of copper. While the 300 mg·kg^−1^ EDTA dose (Treatment 1) resulted in 536.3 mg·kg^−1^ of Cu in the shoots, doubling the chelate dose to 600 mg·kg^−1^ (Treatment 2) significantly increased this accumulation to 1475.8 mg·kg^−1^, representing a nearly threefold enhancement in upward translocation.

Treatment 3 (nZVI application) exhibited a unique influence on the behavior of molybdenum. This treatment promoted the highest aerial accumulation of Mo (52.2 mg·kg^−1^) among all experimental conditions, standing in stark contrast to the negligible amount translocated to the aerial parts in the control group (1.5 mg·kg^−1^).

Correspondingly, the analysis of the remaining substrate (Table 3) reflects these uptake dynamics. For instance, the tailings from Treatments 1 and 2 showed significant depletions in copper and lead compared to the control, directly mirroring the hyperaccumulation observed in the root systems of the EDTA-treated plants.

Remarkably, *Cistanthe grandiflora* successfully survived in a substrate with an electrical conductivity of 34.24 dS·m^−1^, classifying it as a highly salt-tolerant species in addition to its demonstrated metallophytic capabilities.

## 4. Discussion

**Figure 2:**
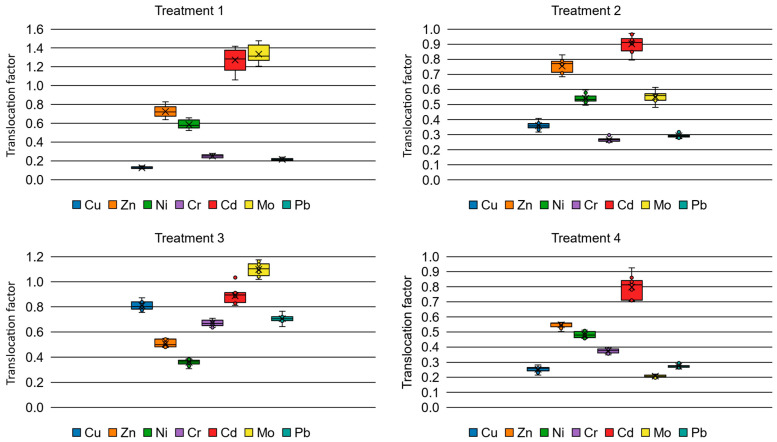
Translocation factors for the different experimental treatments.

The Translocation Factor (TF) was calculated to evaluate the plant’s efficiency in transferring metals from the roots to the shoots under four distinct treatments. The factors were determined after a six-month growth period for each treatment, and the resulting values are presented in Figure 2.

Treatment 1 (300 mg·kg^−1^ EDTA) exhibited the highest translocation potential for Cd and Mo, representing the only experimental condition where TF values for these elements consistently exceeded the 1.0 threshold, reaching peak values of approximately 1.3 for Cd and 1.35 for Mo. This suggests that a moderate dose of EDTA effectively enhances the root-to-shoot transport mechanisms for these specific elements, favoring a phytoextraction strategy. However, this treatment showed limited efficacy for Cu, Pb, and Cr, with TF values remaining below 0.25. This differential mobility can be attributed to the varying stability constants of the different metal–EDTA complexes formed in the rhizosphere, as well as the selective permeability of the root endodermis to these specific chelates.

Compared to Treatment 1, doubling the chelating agent to 600 mg kg^−1^ (Treatment 2) resulted in a general decrease in metal mobility. Notably, the TF for Mo dropped significantly below the 1.0 threshold, while Cd values remained close to unity. This dose-dependent inhibition highlights a well-documented paradox in chelate-assisted phytoremediation: while higher EDTA concentrations maximize metal solubility in the soil, they can also induce severe phytotoxicity. For example, the study of Sun et al. [48] with *S. nigrum* reported the effect of EDTA on Cd uptake, where only a moderate dose of EDTA enhanced its phytoextraction from soil, and a higher dose adversely affected plant growth. Kamal et al. [49] demonstrated that in *Brassica juncea*, EDTA concentrations above 3 mM/kg significantly reduce plant biomass (both roots and shoots) due to acute metal hyperaccumulation, affecting the physiological processes required to pump metals through the xylem. In summary, while ethylenediaminetetraacetic acid (EDTA) is widely utilized to enhance heavy metal bioavailability in soils, the contemporary literature demonstrates that exceeding optimal concentrations is counterproductive for overall phytoextraction efficiency and the Translocation Factor (TF).

Treatment 3 (nZVI) displayed a distinct behavior regarding immobile metals. It yielded the highest observed TF values across all treatments for Cu, Cr, and Pb, averaging 0.80, 0.65, and 0.71, respectively. While nZVI is primarily recognized as an immobilizing agent, its application likely alleviated the severe heavy metal toxicity and oxidative stress at the root interface. By reducing the immediate chemical stress in the rhizosphere, nZVI may have preserved the physiological integrity of the root transport channels, allowing for a greater fraction of the absorbed Cu, Cr, and Pb to be naturally translocated compared to the highly stressed control plants [50].

For copper, the most abundant element in the substrate, the analysis revealed statistically significant differences, enabling a direct comparison across all treatments. In this specific case, the application of nanoparticles (Treatment 3) elevated the TF value, though it remained below 1.0. For chromium, nZVI application doubled the TF value; yet, this enhancement was insufficient to push the parameter above unity. For nickel and zinc, neither the various EDTA doses nor the nanoparticle treatment successfully improved the translocation factor significantly.

The results obtained from the control group (Treatment 4) indicate that *Cistanthe grandiflora* naturally behaves as a robust metal-excluder species regarding copper and other heavy metals. Although the substrate contained extreme concentrations of copper (~4513 mg·kg^−1^), the accumulation in the aerial parts remained relatively low compared to the massive sequestration observed in the roots. The TF values for Cu, Zn, and Pb were consistently below 1.0 across most treatments, suggesting that the plant’s primary evolutionary defense mechanism in metalliferous environments involves sequestering toxic ions in the root, thereby restricting their transport to photosynthetic tissues.

**Figure 3:**
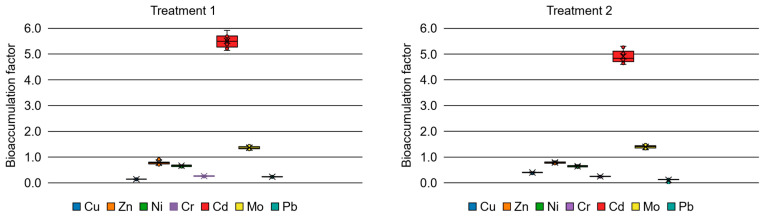

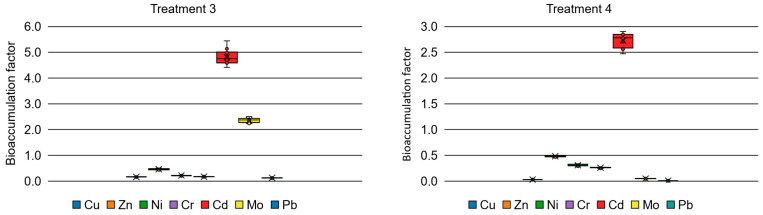
Bioaccumulation factors for the different experimental treatments.

**Figure 4:**
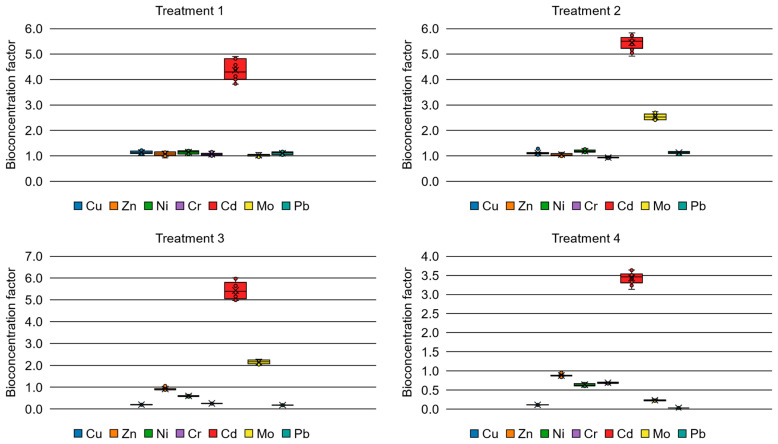
Bioconcentration factors for different experimental treatments.

Contrary to initial expectations regarding total uptake, the application of zero-valent iron nanoparticles (nZVI) did not result in a statistically significant improvement in overall metal bioaccumulation compared to the 300 mg·kg^−1^ EDTA dose. To further evaluate the specific phytoremediation pathways, the Bioaccumulation Factor (BAF) and Bioconcentration Factor (BCF) were calculated under the same experimental conditions and are presented in Figure 3 and Figure 4.

A major finding of this study is the behavior of cadmium, which was the only element that demonstrated a significant increase in BAF across all treatments. Remarkably, even without amendments, the BAF for Cd was approximately 2.7. This indicates an inherent, strong natural tendency of *Cistanthe grandiflora* to bioaccumulate Cd in its shoots, highlighting the species as a natural accumulator for this highly toxic metal regardless of external chemical assistance.

Molybdenum showed a distinct response to the chemical amendments. In the control group, the BAF was close to zero (below 0.2), indicating that the plant does not naturally accumulate Mo in its aerial tissues. However, the application of amendments successfully pushed the BAF above the 1.0 threshold. Treatment 3 resulted in the highest BAF for Mo (approx. 2.2), followed by Treatment 2 (approx. 1.4). Consequently, while the plant naturally acts as an excluder for Mo, the amendments—particularly nZVI—effectively altered the soil chemistry to induce its bioaccumulation in the shoots.

For the primary tailings contaminant (copper), as well as zinc and lead, the BAF values remained consistently low (below 1.0) across all treatments, reaffirming the phytostabilization tendency.

Regarding the Bioconcentration Factor (BCF), the addition of EDTA produced a significant, transformative increase for both copper and nickel. In the control group, BCF values were 0.11 and 0.60, respectively; these surged to approximately 1.1 following EDTA application. This effect was consistent regardless of the specific EDTA dose. Conversely, the addition of nanoparticles had no discernible effect on the BCF for these specific metals, aligning with nZVI’s primary mechanism of precipitating and immobilizing metals in the bulk soil rather than facilitating root entry.

For molybdenum, all treatments increased the BCF value. This increment was particularly notable with the 600 mg·kg^−1^ EDTA dose, where the mean BCF surged from 0.23 in the control group to 2.64. Similarly, the BCF for lead showed a highly significant increase with both EDTA doses, rising from a baseline value of approximately 0.03 to 1.1.

Overall, the application of EDTA proved to be the most influential factor in altering metal mobility within the root zone. Consistent with previous studies on chelate-assisted phytoremediation [51,52], the addition of EDTA significantly increased the BCF for Cu, Ni, Pb, and Mo, raising values from less than 0.5 in the control to over 1.0 in treated plants. This enhancement is attributed to the formation of soluble metal–EDTA complexes, which overcome the natural insolubility of these metals in the alkaline tailings (pH 6.7), thereby facilitating their considerable uptake by the root system [53]. Notably, the concentration of copper in the roots increased nearly tenfold compared to the control, reaching elevated sequestration values over 4000 mg·kg^−1^.

However, it is crucial to emphasize the environmental trade-offs: while EDTA successfully enhanced root uptake and stabilization capabilities, the creation of highly mobile metal complexes simultaneously poses a significant risk of groundwater contamination via leaching. This dictates that any potential field-scale application of EDTA-assisted phytoremediation in these tailings requires rigorous hydrological monitoring and strict dosage control.

## 5. Conclusions

This study evaluated the potential of the native species *Cistanthe grandiflora* for the phytoremediation of copper mine tailings. *Cistanthe grandiflora* demonstrates a robust tolerance to heavy metal stress and functions primarily as a phytostabilizer. It effectively accumulates metals within its root system while restricting their translocation to aerial tissues, a characteristic highly suitable for reducing the bioavailability of contaminants in arid mining zones.

The application of EDTA significantly enhanced the plant’s extraction capacity. It successfully increased the Bioconcentration Factor (BCF) for Cu, Ni, and Pb to values greater than 1.0. Furthermore, the lower EDTA (300 mg·kg^−1^) proved sufficient to elevate the Translocation Factor of cadmium, suggesting a targeted potential for the phytoextraction of this specific element.

Under the tested conditions, the application of nZVI demonstrated limited efficacy in improving the evaluated phytoremediation factors. Ultimately, the inherent metallophytic traits of *Cistanthe grandiflora*, when enhanced by the controlled application of EDTA, represent a promising and viable strategy for the stabilization of mine tailings in northern Chile.

## Figures and Tables

**Table 1:** Characteristics of the substrate (tailings).

Parameter	Unit	Concentration in mg·kg^−1^
Cu	mg·kg^−1^	4513.01 ± 91.14
Zn	mg·kg^−1^	1147.56 ± 31.54
Ni	mg·kg^−1^	91.77 ± 5.61
Cr	mg·kg^−1^	214.17 ± 7.72
Cd	mg·kg^−1^	7.56 ± 0.38
Mo	mg·kg^−1^	68.34 ± 2.45
Pb	mg·kg^−1^	674.27 ± 8.56
Specific gravity	-	2.83 ± 0.33
pH	-	7.50 ± 0.20
Solid concentration	Weight %	87.00 ± 1.2
Granulometry d_50_	μm	0.05 ± 0.002
Electric conductivity	dS·m^−1^ at 25 °C	34.24 ± 0.15
Calcium percentage as CaO	% *w*/*w*	9.55 ± 0.32
Magnesium percentage as MgO	% *w*/*w*	5.10 ± 0.17
Manganese percentage as MnO	% *w*/*w*	0.25 ± 0.01
Sodium percentage as Na_2_O	% *w*/*w*	2.41 ± 0.07
Potassium percentage as K_2_O	% *w*/*w*	2.10 ± 0.09
Phosphorus percentage as P_2_O_5_	% *w*/*w*	0.17 ± 0.03

**Table 2:** Final metal concentrations (mg·kg^−1^ dry weight) in plant tissues (aerial parts and roots) after the six-month experimental period.

mg·kg^−1^ Dry Substrate	Cu	Zn	Ni	Cr	Cd	Mo	Pb
Treatment 1 aerial	536.3 ± 27.2 ^c^	741.9 ± 36.3 ^a^	22.2 ± 0.9 ^a^	29.6 ± 1.4 ^b^	20.9 ± 2.9 ^a^	30.1 ± 1.2 ^b^	140.6 ± 3.9 ^b^
Treatment 1 roots	4246.4 ± 211.5 ^a^	1025.9 ± 49.1 ^a^	38.1 ± 1.9 ^a^	118.5 ± 5.1 ^a^	15.9 ± 1.2 ^b^	22.6 ± 1.1 ^c^	652.6 ±32.2 ^a^
Treatment 2 aerial	1475.8 ± 55.0 ^a^	769.7 ± 37.3 ^a^	21.0 ±0.9 ^a^	28.5 ± 1.3 ^b^	18.8 ± 0.9 ^ab^	30.2 ± 1.3 ^b^	195.0 ± 8.1 ^a^
Treatment 2 roots	4116.6 ± 202.4 ^a^	1021.1 ± 49.3 ^a^	39.0 ± 1.5 ^a^	107.0 ± 5.2 ^a^	20.8 ± 1.0 ^a^	54.9 ± 2.3 ^a^	665.8 ± 25.7 ^a^
Treatment 3 aerial	636.8 ± 21.3 ^b^	409.1 ± 15.8 ^c^	7.1 ± 0.3 ^c^	20.9 ± 1.0 ^c^	17.3 ± 0.7 ^b^	52.2 ± 1.7 ^a^	73.7 ± 42.5 ^c^
Treatment 3 roots	786.3 ± 23.8 ^b^	808.5 ± 28.4 ^c^	19.8 ± 1.0 ^c^	31.2 ± 1.2 ^b^	19.5 ± 0.7 ^a^	47.6 ± 1.7 ^b^	104.6 ± 5.1 ^d^
Treatment 4 aerial	119.8 ± 4.5 ^d^	512.0 ± 4.1 ^b^	16.5 ± 0.5 ^b^	43.3 ± 1.8 ^a^	14.9 ± 0.8 ^c^	1.5 ± 0.2 ^c^	5.1 ± 0.2 ^d^
Treatment 4 roots	470.8 ± 18.1 ^c^	929.1 ± 25.9 ^b^	35.1 ± 3.8 ^b^	114.1 ± 3.3 ^a^	18.7 ± 0.8 ^a^	7.7 ± 0.3 ^d^	18.4 ± 3.1 ^c^

Note: The comparison of means was performed separately for aerial parts and roots for each metal. Values followed by different letters within the same column and tissue type indicate significant differences according to Tukey’s test (*p* < 0.05).

**Table 3:** Final metal concentrations (mg·kg^−1^ dry substrate) in the tailings after the six-month experimental period.

mg·kg^−1^ Dry Substrate	Cu	Zn	Ni	Cr	Cd	Mo	Pb
Treatment 1	3758.0 ± 85.7 ^c^	954.0 ± 52.6 ^b^	33.3 ± 0.7 ^b^	111.5 ± 3.1 ^c^	3.6 ± 0.1 ^b^	22.0 ± 0.4 ^b^	590.8 ± 7.5 ^b^
Treatment 2	3700.4 ± 40.3 ^c^	972.6 ± 15.6 ^b^	32.9 ± 0.4 ^b^	114.6 ± 4.6 ^c^	3.8 ± 0.1 ^b^	21.6 ± 0.4 ^b^	591.0 ± 1.5 ^b^
Treatment 3	3963.2 ± 41.3 ^b^	887.6 ± 29.2 ^c^	33.0 ± 0.6 ^b^	122.4 ± 0.7 ^b^	5.5 ± 0.2 ^a^	22.1 ± 0.5 ^b^	590.7 ± 15.6 ^b^
Treatment 4	4173.7 ± 37.8 ^a^	1059.0 ± 18.3 ^a^	54.6 ± 2.7 ^a^	165.9 ± 4.5 ^a^	5.5 ± 0.1 ^a^	32.5 ± 1.5 ^a^	610.0 ± 1.4 ^a^

Note: Values followed by different letters within the same column indicate significant differences according to Tukey’s test (*p* < 0.05).

## Data Availability

The datasets generated during the current study are available in the Kaggle repository, https://www.kaggle.com/datasets/andrealazo/fitorremediation-data (accessed on 9 February 2026).
